# The relationship between comorbid overweight-obesity and cold executive functions, verbal short-term memory, and learning in attention deficit hyperactivity disorder

**DOI:** 10.1192/j.eurpsy.2021.1695

**Published:** 2021-08-13

**Authors:** H.A. Guler, S. Turkoglu

**Affiliations:** 1 Department Of Child And Adolescent Psychiatry, Afyonkarahisar State Hospital, Afyonkarahisar, Turkey; 2 Department Of Child And Adolescent Psychiatry, Selcuk University Medicine Faculty, Konya, Turkey

**Keywords:** Executive functions, obesity, learning, attention deficit hyperactivity disorder

## Abstract

**Introduction:**

Attention deficit hyperactivity disorder (ADHD) is the most common neurodevelopmental disorder in childhood. ADHD is a risk factor for the development of overweight and obesity. One neuropsychological factor that may play a prominent role in the relationship between ADHD and obesity is executive functioning.

**Objectives:**

The aim of this study is to investigate the relationship between comorbid obesity/overweight and cold executive functions, verbal short-term memory, and learning in children with ADHD. This is the first study to examine relationship between verbal short-term memory-learning and obesity in patients with ADHD.

**Methods:**

This study was conducted with 70 patients with ADHD and 30 healthy controls. In this study, patients diagnosed with ADHD were divided into two groups according to body mass index (BMI) as <85 percentile and ≥85 percentile. Cold executive functions were evaluated by Stroop Test (ST) and Cancellation Test (CT). Serial Digit Learning Test (SDLT) was administered to measure verbal short-term memory and learning capasity. In order to evaluate the severity of ADHD objectively, parents completed the Conners’ Parents Rating Scale-Revised Short Version (CPRS-RS).

**Results:**

The ST, SDLT and CT scores were significantly lower in both groups with ADHD than the control group. The CPRS-RS subscale scores were significantly higher in both groups with ADHD than the control group. There was no statistically significant difference in ST, SDLT, CT scores and CPRS-RS subscale scores between the two groups with ADHD.

**Conclusions:**

This study show that overweight/obesity comorbid with ADHD was not associated with cold executive functions, verbal short-term memory, learning, or ADHD symptom severity.

**Disclosure:**

No significant relationships.

